# Therapeutic Alliance and Treatment Outcome in Cognitive Behavior Therapy for Obsessive-Compulsive Disorder

**DOI:** 10.3389/fpsyt.2022.658693

**Published:** 2022-03-24

**Authors:** Nadja Wolf, Patricia van Oppen, Adriaan W. Hoogendoorn, Anton J. L. M. van Balkom, Henny A. D. Visser

**Affiliations:** ^1^Mental Health Care Institute Geestelijke gezondheidszorg (GGZ) Centraal, Amersfoort, Netherlands; ^2^Department of Psychiatry, Amsterdam Public Health, Amsterdam University Medical Center, Vrije Universiteit, Amsterdam, Netherlands; ^3^Geestelijke Gezondheidszorg (GGZ) InGeest Specialized Mental Health Care, Amsterdam, Netherlands

**Keywords:** therapeutic alliance, Obsessive-Compulsive Disorder (OCD), psychotherapy outcome, predictor, Cognitive-Behavioral Therapy (CBT)

## Abstract

**Objective:**

Therapeutic alliance has consistently been found to predict treatment outcomes across various psychotherapies and patient diagnosis. However, the relationship between therapeutic alliance and outcome in Cognitive Behavioral Therapy (CBT) has shown mixed results. This study investigated the impact of different aspects of therapeutic alliance in CBT for Obsessive-Compulsive Disorder (OCD).

**Method:**

Data from two previously completed randomized controlled trials of 208 patients with OCD and their therapists were analyzed. Therapeutic alliance was assessed at week 4 of treatment with the patient-rated and therapist-rated Working Alliance Inventory (WAI), which includes three subscales to measure alliance domains (Goal, Task and Bond). Higher WAI score reflects a better therapeutic relationship. OCD severity was rated by independent assessors at baseline and post-treatment using the Yale-Brown Obsessive-Compulsive Scale (Y-BOCS). Linear regression analyses were used to examine the effects of the different aspects of therapeutic alliance on treatment outcome, adjusted for baseline symptom severity.

**Results:**

A higher total WAI score as rated by therapists significantly predicted a lower post-treatment Y-BOCS. Further, higher scores on the Goal and Task subscales of the WAI were associated with lower post-treatment severity. However, these significant outcomes reflected only small effect sizes.

**Conclusions:**

In the treatment of OCD, the strength of the therapeutic alliance contributes to outcomes, though to a limited extent. Effective OCD treatment involves the delivery of specific therapy interventions, in the context of a strong therapeutic alliance.

## Introduction

A considerable amount of research has been directed toward the role of specific factors vs. common factors in predicting psychotherapeutic outcome. Specific factors refer to techniques or interventions that are specific to a particular treatment orientation, whereas common factors are elements of psychotherapy that are shared by all treatment approaches or models (e.g. therapeutic alliance and expectations). There is an ongoing debate about the relative importance of both factors in producing therapeutic change. Many researchers have argued that common factors contribute more to treatment outcome than the specific factors [e.g. (1, 2)], however, this contention is challenged by others [e.g. (3, 4)]. Nevertheless, one common factor has consistently emerged as a moderate predictor of outcome: therapeutic alliance. Several large meta-analyses have repeatedly shown associations between therapeutic alliance and treatment outcome in a wide range of disorders and psychotherapies, accounting for ~5–8% of the total variance in outcome [e.g. (5–7)].

Therapeutic alliance has been defined as a combination of an affective bond between patient and therapist and their collaboration at achieving agreed-upon therapy tasks and goals (8).

Although it was initially a psychoanalytic construct [e.g. (9)], it has become a focus of interest across different treatment modalities, including Cognitive Behavioral Therapy (CBT). In CBT, a solid therapeutic alliance is viewed as necessary, but not sufficient on itself, to promote change (10). Results of previous research in the field of CBT on the alliance-outcome association are inconsistent. Multiple studies have shown that a stronger therapeutic alliance in CBT predicts better treatment outcome [e.g. (11, 12)], while others have found little or no alliance-outcome associations [e.g. (13, 14)]. One possible cause for heterogeneity across these studies might be the potentially moderating effect of patient diagnoses; that is, the impact of therapeutic alliance on treatment outcomes might vary by diagnostic group (5).

While many studies have investigated therapeutic alliance and CBT outcomes across a variety of patient diagnoses, only a few have investigated its impact on treatment outcomes of OCD, and with mixed results. Strauss (15) completed a trial to assess the relative contribution of common factors, including therapeutic alliance, vs. specific factors in the treatment of OCD and found that therapeutic alliance did not account for significant variance in treatment outcome. He concluded that in the context of CBT for OCD specific treatment factors matter more than common factors. Wheaton (16) also found that overall therapeutic alliance was not related to OCD treatment outcome. In contrast, other previous studies have suggested that a stronger patient-rated therapeutic alliance was associated with a larger improvement in post-treatment OCD symptom severity (12, 17–20). These inconsistent findings might be a reflection of the different ways in which therapeutic alliance was assessed across these studies (e.g. different rating scales and early- vs. mid-treatment assessment).

Most therapeutic alliance studies into OCD treatment outcomes have focused on the predictive value of patient ratings, while little attention has been paid to the predictive value of ratings by therapists. Therapist ratings have been found to predict OCD treatment outcomes in only one study (17), while others found no significant relationship (15, 18). Further, little is known about which domains of therapeutic alliance relate most to OCD treatment outcome. Therapeutic alliance is commonly seen as having three domains (8), including patient's and therapist's agreement on therapeutic goals (Goal alliance), their agreement on tasks in treatment (Task alliance), and the development of bonds between the patient and therapist (Bond alliance). The individual impact of each of these domains on outcome may differ, which for example was shown by Webb et al. (21), who reported that Task and Goal alliance were related to outcome in the treatment of depression with Cognitive Therapy, whereas Bond alliance was not. To date, only two studies have published data on the predictive value of the individual domains of therapeutic alliance in the treatment of OCD. One study found that better treatment outcomes were predicted by higher ratings on the Task alliance, but not Bond or Goal alliance (16). Another study showed that Task and Goal alliance were predictive for OCD symptom severity post-treatment, whereas Bond alliance was not (22). Thus, several aspects of therapeutic alliance in OCD treatment remain relatively unexplored.

The aim of the current study is to replicate and extend previous research on the relationship between therapeutic alliance and treatment outcome in patients with OCD. If quality of therapeutic alliance is an important factor in OCD treatment, interventions to improve the therapeutic alliance may maximize therapeutic outcomes. On the other hand, if the predictive value of therapeutic alliance in OCD treatment is limited, one may focus more on therapy specific interventions to enhance treatment outcomes. The first objective is to determine to what extent overall therapeutic alliance rated by patients as well as therapists contributes to treatment outcome in patients with OCD. The second objective is to explore the individual predictive value of the different domains of therapeutic alliance on OCD treatment outcome.

## Materials and Methods

### Design and Participants

The data for the present study were selected of two previously completed trials (23, 24), who both had unanalyzed data on therapeutic alliance and treatment outcome. The first trial was a randomized controlled trial (RCT) previously described by Van Oppen (23), from now on referred to as the Exposure with Response Prevention-RCT (EX/RP-RCT). The aim of this study was to compare the effectiveness of four different modes of delivery of EX/RP (i.e. therapist-guided or self-guided EX/RP performed by experienced behavior therapists or by master's students of clinical psychology) (*N* = 118). The study was conducted between January 1999 and January 2005, at the academic outpatient clinic of a mental health institute specialized in anxiety disorders. Eligible participants were 18 years of age or older and had a main diagnosis of OCD according to the DSM-IV, with at least a 1-year duration at intake. Criteria for excluding participants were: OCD with obsessions only, suicidal intents, organic brain disease, past or present psychosis, psychoactive substance use disorder, severe borderline or antisocial personality disorder. All DSM-IV Axis I disorders were confirmed with the SCID-I (25). In addition, participants were excluded if they were concomitantly being treated elsewhere, had been treated with behavior or cognitive therapy in the 6 months preceding baseline or were using benzodiazepines in a dose of more than 15 mg diazepam equivalents per day. Patients taking psychoactive drugs were included if they were willing and able to discontinue the use of medication at least 4 weeks prior to randomization. Approval of the study was granted by the VU-University Medical Centre's Ethical Review Committee, Amsterdam. Demographic and clinical characteristics at baseline did not differ significantly across groups. Further, no significant differences were found in post-treatment symptom severity between any of the groups. Full study details are provided elsewhere (23).

The second trial was a multicenter RCT designed to study the superiority of the Inference Based Approach (IBA) compared to CBT in patients with OCD with poor insight (*N* = 90) (24), from now on referred to as the IBA-RCT. This study was conducted at three specialized anxiety disorder departments of mental health care institutions in the Netherlands. Recruitment took place between January 2009 and March 2012. Eligible participants were adults (aged 18 or older) with a primary diagnosis of OCD with poor insight according to the DSM-IV criteria, with a Yale-Brown Obsessive-Compulsive Scale (Y-BOCS) score of at least 16, and who either were not on psychotropic medication, or had been taking stable doses for at least 8 weeks before baseline measure and agreed to maintain dosage levels unchanged during the study. Exclusion criteria were a psychotic disorder, an organic mental disorder, substance dependence, a pervasive developmental disorder, mental retardation or insufficient comprehension of the Dutch language. These in- and exclusion criteria were assessed with The Structured Clinical Interview for DSM-IV-TR (SCID-I) (26). This study was approved by the medical ethics review board METiGG (Utrecht, the Netherlands). There were no statistically significant differences between the two treatment groups at baseline. In addition, no significant difference was found in clinical outcome between treatment conditions. A detailed description of the sample characteristics and methodology of the study are described elsewhere (24).

It was thought justifiable to combine the data from the two trials for the purpose of this study, since participants from both trials were treated with cognitive behavior therapies for OCD, at specialized anxiety clinics. Furthermore, both trials assessed therapeutic alliance from a patient's and therapist's perspective at the same time in treatment, with the same instrument. The statistical justification to combine the data of the two RCT's is presented under the Results section (under “Sample description”).

The present study is registered at the Open Science Foundation.

### Treatments and Therapists

Participants from both trials were treated using cognitive behavior therapies; participants in the EX/RP-RCT were treated with either self-guided or therapist-guided EX/RP, and participants in the IBA-RCT were treated with CBT consisting of CT and self-guided EX/RP or with IBA. These treatment conditions emphasize different aspects of the obsessional chain; IBA focuses on the process preceding obsessional development, whereas CBT targets the processes following the occurrence of obsessions (such as the appraisals of the obsessions with CT and compulsions with EX/RP) (27). Although the treatment conditions differ in their focus on the obsessional chain, they are all focused forms of psychotherapy for OCD. All treatments were delivered once a week and standardized session-by-session protocols with standardized forms for exercises and homework assignments were used. The sessions of all treatment conditions followed a standard format, with agenda setting, evaluating homework assignments, in-session practicing and evaluating exercises and planning new homework. All treatment conditions teach patients how to defend themselves against the automatic performance of compulsions and against the absorbing effect of obsessive thoughts.

In the EX/RP-RCT a total of 19 therapists participated; six experienced behavior therapists (four woman, two men) and 13 master's clinical psychology students (10 women, three men). Experienced behavior therapists had a master's degree in clinical psychology, had completed a postgraduate CBT training, and had ~15 years of experience with the treatment of OCD. Master's clinical psychology students were in their final year of completing their master's degree and did not have any experience in providing behavior therapy. Students received a 2-day EX/RP workshop. All therapists received weekly supervision.

In the IBA-RCT a total of 23 therapists participated; nine therapists provided IBA (seven women, two men) and 14 therapists provided CBT (13 women, one man). All therapists had at least two years of experience with the treatment of OCD with CBT; most of them were licensed as CBT therapists. Therapists who provided IBA were trained in a 5-day IBA workshop and treated at least two patients as a training exercise prior to the beginning of the study. Therapists had weekly supervision for six months, then once every two weeks.

### Measurements

#### Therapeutic Alliance

The Working Alliance Inventory [WAI; (28)] was used to assess therapeutic alliance. The WAI is one of the most commonly used questionnaires to measure therapeutic alliance [e.g. (5)]. It is developed as a 36-item questionnaire based on Bordin's concept measuring three domains: Bond (12 items, e.g. “I feel my therapist appreciates me”), Task (12 items, e.g. “my therapist and I agree about the things I will need to do in therapy to help improve my situation”), and Goal (12 items, e.g. “my therapist and I are working toward mutually agreed upon goals”). The WAI consists of parallel patient (WAI-c) and therapist (WAI-t) versions (e.g. WAI-c: “I feel my therapist appreciates me” vs. WAI-t: “I feel that my patient appreciates me”). For the present study, the Dutch version of the WAI was used (29). A 5-point Likert scale ranging from “never” to “always” was used to rate each item. WAI scores range from 36 to 180, with higher scores indicating stronger therapeutic alliance. For both the patient and therapist ratings, a mean score was calculated if at least 75% of the items had been completed. The WAI-c and WAI-t were rated at week 4 of the treatment (after session 4). The WAI has shown good to excellent scale reliability for the patient (estimates of Cronbach's α = 0.93) and therapist (estimates of Cronbach's α = 0.87) versions (28, 30). In the present study, total scores on the WAI demonstrated excellent internal consistency (WAI-c α = 0.91, WAI-t α = 0.94). The subscales demonstrated acceptable to excellent internal consistency (WAI-c: Goal: α = 0.83, Task: α = 0.87, Bond: α = 0.90; WAI-t: Goal: α = 0.83, Task: α = 0.82, Bond: α = 0.73).

#### OCD Symptom Severity

The clinician-rated Yale-Brown Obsessive-Compulsive Scale (Y-BOCS) was used to assess OCD symptom severity. The Y-BOCS is regarded as the gold standard instrument for the measurement of OCD severity (31, 32). This is a 10-item scale assessing the severity of obsessions and compulsions in the past week, with total scores ranging from 0 (no symptoms) to 40 (extreme symptoms). The Y-BOCS severity scale has well-documented validity and reliability (31, 32). The internal consistency in the present sample was α = 0.77. The Y-BOCS was assessed at baseline and post-treatment by independent and blind research assessors who were trained, monitored and supervised in the assessment technique.

### Statistical Analyses

Descriptive statistics and percentages were calculated for demographic and psychometric variables. *T*-tests and chi-square test were used to assess group differences between participants who participated in the EX/RP-RCT and the IBA-RCT. *T*-test was used to compare therapeutic alliance ratings between participants who completed and did not complete treatment. Pearson's correlation coefficients were calculated to evaluate the relationship between patient-rated and therapist-rated therapeutic alliance.

A linear regression analysis was used to determine whether overall therapeutic alliance (i.e., WAI score) predicted post-treatment OCD severity (i.e., Y-BOCS score) adjusted for pretreatment severity. Further, three separate linear regression analyses were conducted to test the associations between the subscales of the WAI (Goal, Task, Bond) and treatment outcome. Subsequently, if one or more of the subscales showed a clinically meaningful effect, the independent effects of the subscales would be assessed using multiple regression analyses that included all three subscales as independent variables. All analyses were conducted for patient-rated and therapist-rated therapeutic alliance scores separately.

Both a complete case analysis as well as an analysis using multiple imputation to include all participants (an intention-to-treat analysis) were carried out and the significance level was set a *p* < 0.05. Studying an existing sample consisting of 208 patients gave us sufficient power (0.80) to detect a small to medium effect size—in terms of (an increase in) *R*^2^ = 0.037 or higher—using linear regression to study effects on the change in OCD severity with an alpha 0.05. Statistical analyses were performed using SPSS software (version 25).

## Results

### Sample Description

The sample consisted of 208 participants with an average age of 34.9 years (SD 10.2), who were predominantly female (62.5%). Table 1 provides the means and standard deviations for the outcome variable and each predictor at baseline. The mean Y-BOCS score at baseline was 26.15 (SD 5.1), reflecting a general severe OCD symptom severity. The sample mean on the total WAI score was 152.80 (SD 17.5, range = 106–180, with one outlier of 51) for patients, and 144.52 (SD 13.0, range =101–171) for therapists. Thus, results indicated that both patients and therapists generally experienced a strong therapeutic alliance. Therapist and patient total WAI scores correlated weakly positive (*r* = 0.16, *p* = *0.04)*, indicating a low level of agreement between patients and therapists on the strength of therapeutic alliance. The patient-rated WAI subscales were moderately to highly inter-correlated, as were the therapist-rated WAI subscales (see Table 2).

**Table 1:** Demographic and clinical characteristics of the study sample.

	**Total**	**EX/RP-RCT**	**IBA-RCT**	**p^**a**^**
**Demographics**	*N* = 208	*N* = 118	*N* = 90	
Age (years)	34.9 ± 10.2	35.1 ± 10.7	34.7 ± 9.5	0.805
Female, no. (%)	130 (62.5)	71 (60.2)	59 (65.6)	0.427
**Clinical characteristics**				
Baseline Y-BOCS	26.15 ± 5.1	26.25 ± 5.5	26.02 ± 4.6	0.756
	*N* = 187	*N* = 108	*N* = 79	
Post-treatment	16.27 ± 8.0	16.09 ± 8,3	16.51 ± 7.6	0.729
Y-BOCS				
**WAI patient**	*N* = 174	*N* = 101	*N* = 73	
WAI-c Total	152.80 ± 17.5	150.32 ± 19.8	156.23 ± 12.9	**0.018**
WAI-c Goal	50.42 ± 6.5	49.74 ± 7.5	51.35 ± 4.8	0.086
WAI-c Task	50.75 ± 6.5	50.08 ± 7.2	51.57 ± 5.3	0.093
WAI-c Bond	51.66 ± 6.4	50.53 ± 7.1	53.21 ± 5.0	**0.004**
**WAI therapist**	*N* = 191	*N* = 108	*N* = 83	
WAI-t Total	144.52 ± 13.0	147.03 ± 13.9	141.25 ± 11.0	**0.002**
WAI-t Goal	47.91 ± 5.5	49.17 ± 5.7	46.27 ± 4.8	**<0.001**
WAI-t Task	48.75 ± 5.2	49.88 ± 5.5	47.29 ± 4.5	**0.001**
WAI-t Bond	47.86 ± 4.3	47.98 ± 4.8	47.70 ± 3.5	0.640

*Values are represented as mean ± SD*.

a *Comparison between EX/RP-RCT and IBA-RCT using chi-square likelihood ratio statistics for categorical variables and t-test for continuous variables*.

*Bold indicates p < 0.05*.

*EX/RP, Exposure with Response Prevention; RCT, randomized controlled trial; IBA, Inference Based Approach; Y-BOCS, Yale-Brown Obsessive-Compulsive Scale; WAI-c, Working Alliance Inventory-client; WAI-t, Working Alliance Inventory-therapist; SD, standard deviation*.

**Table 2:** Pearson correlations.

		**2**	**3**	**4**	**5**	**6**	**7**	**8**
1	WAI-c Total	0.92^**^	0.91^**^	0.87^**^	0.16^*^	0.16^*^	0.19^*^	0.06
2	WAI-c Goal	^*^	0.79^**^	0.69^**^	0.15^*^	0.15^*^	0.19^*^	0.05
3	WAI-c Task		^*^	0.67^**^	0.21^**^	0.22^**^	0.24^**^	0.06
4	WAI-c Bond			^*^	0.08	0.06	0.08	0.07
5	WAI-t Total				^*^	0.91^**^	0.91^**^	0.75^**^
6	WAI-t Goal					^*^	0.80^**^	0.51^**^
7	WAI-t Task						^*^	0.52^**^
8	WAI-t Bond							^*^

*WAI-c, Working Alliance Inventory-client; WAI-t, Working Alliance Inventory-therapist*.

* *p < 0.05*.

** *p < 0.01*.

When comparing the participants from the EX/RP-RCT with participants from the IBA-RCT, no significant differences were found for age, gender and baseline Y-BOCS. However, significant differences were found for the mean total WAI score for patients, which was significantly higher in the IBA-RCT group [*t*_(170)_ = 2.38, *p* = 0.018], and the mean total WAI score for therapists, which was significantly higher in the EX/RP-RCT group [*t*_(189)_ = −3.11, *p* = 0.002]. To adjust for these differences, group membership of the RCT was entered as a covariate in all further analyses.

When comparing participants who completed (*N* = 187) and did not complete treatment (*N* = 21, of whom 10 withdrew before assessment of therapeutic alliance), group differences for the mean total WAI scores as rated by patients or therapists were not statistically significant (*p* > 0.05).

Analyses using complete-cases and analyses using multiple imputation showed similar results in all analyses mentioned below. Given the limited number of missing values, findings from the complete-cases analyses are presented in the sections below.

### Predictive Value of Overall Therapeutic Alliance on OCD Treatment Outcome

Total therapeutic alliance scores as rated by therapists (WAI-t: *b* = −0.10, 95% CI = [−0.19, −0.01], *p* = 0.028, ΔR^2^ = 0.023) statistically significantly predicted post-treatment OCD severity, whereas total therapeutic alliance scores as rated by patients approached statistical significance (WAI-c: *b* = −0.07, 95% CI= [−0.14, 0.00], *p* = 0.050, ΔR^2^ = 0.020) (see Table 3). These results show that an increase of 10 points in therapist-WAI-scores corresponds to only 1 point decrease in post-treatment Y-BOCS (range 0–40), while the strength of the therapeutic alliance in our sample was high with a mean score of 144.5 of a maximum score of 180. Thus, a very large increase in strength of therapeutic alliance is needed for a clinically significant decrease in post-treatment symptom severity. This suggests that therapist-rated therapeutic alliance, albeit statistically related to treatment outcome, as well as patient-rated therapeutic alliance are not clinically relevant predictors of post-treatment symptom severity.

**Table 3:** Results from two separate linear regression models predicting post-treatment OCD severity from therapeutic alliance (from patient and therapeutic perspective) adjusted for baseline OCD severity and for group membership of the RCT.

**Predictors**	**b**	**95%-CI**	***p*** **-value**	**ΔR^**2**^**
Total WAI-c (*N* = 163)	−0.07	(−0.14, 0.00)	0.050	0.020
Total WAI-t (*N* = 179)	−0.10	(−0.19, −0.01)	**0.028**	0.023

*OCD, Obsessive-Compulsive Disorder; RCT, Randomized Controlled Trial; b, unstandardized regression coefficient; CI, confidence interval; WAI-c, Working Alliance Inventory-client; WAI-t, Working Alliance Inventory-therapist*.

*Bold indicates p < 0.05*.

### Predictive Value of Different Domains of Therapeutic Alliance on OCD Treatment Outcome

The patient-rated Goal and Task subscales of the WAI as well as the therapist-rated Goal subscale significantly predicted post-treatment OCD symptom severity (WAI-c-Goal: *b* = −0.21, 95% CI = [−0.40, −0.03], *p* = 0.021, ΔR^2^ = 0.028; WAI-c-Task: *b* = −0.24, 95% CI = [−0.42, −0.06], *p* = 0.008, ΔR^2^ = 0.037; WAI-t-Goal: *b* = −0.29, 95% CI = [−0.50, −0.08], *p* = 0.008, ΔR^2^ = 0.034), whereas the other subscales of the WAI did not (see Table 4). These results show that an increase of 10 point in a WAI-subscale-score corresponds to 2 to 3 points decrease of posttreatment Y-BOCS, while the means of the subscales were already relatively high with means of approximately 50 of a maximum score of 60. This suggests that none of the different subscales are clinically relevant predictors of post-treatment symptom severity. A multiple regression analysis including all three subscales was not performed, since the effects of the subscales were so small that it was not relevant to assess the independent effects of the subscales.

**Table 4:** Results from six separate linear regression models predicting post-treatment OCD severity from three subscales of therapeutic alliance, i.e. Goal, Task and Bond alliance (from patient and therapeutic perspective) adjusted for baseline OCD severity and for group membership of the RCT.

**Predictors**	**b**	**95%-CI**	***p*** **-value**	**ΔR^**2**^**
**Patients (*****N*** **=** **163)**				
WAI-c Goal	−0.21	(−0.40, −0.03)	**0.021**	0.028
WAI-c Task	−0.24	(−0.42, −0.06)	**0.008**	0.037
WAI-c Bond	−0.03	(−0.22, 0.16)	0.729	0.001
**Therapist (*****N*** **=** **179)**				
WAI-t Goal	−0.29	(−0.50, −0.08)	**0.008**	0.034
WAI-t Task	−0.20	(−0.43, 0.03)	0.083	0.015
WAI-t Bond	−0.15	(−0.42, 0.12)	0.263	0.006

*OCD, Obsessive-Compulsive Disorder; RCT, Randomized Controlled Trial; b, unstandardized regression coefficient; CI, confidence interval; WAI-c, Working Alliance Inventory-client; WAI-t, Working Alliance Inventory-therapist*.

*Bold indicates p < 0.05*.

## Discussion

We studied the contribution of patient-rated and therapist-rated therapeutic alliance in the reduction of OCD symptoms after treatment. In addition, we explored the impact of different aspects of therapeutic alliance on OCD treatment outcome. We may conclude that in the treatment of OCD therapeutic alliance has a limited contribution in predicting therapeutic outcomes.

Our results showed that the overall strength of the therapeutic alliance from a therapist perspective predicted greater improvements in OCD symptoms, however, the predictive effect was small, and clinically not very meaningful. For therapeutic alliance from a patients perspective we failed to find evidence that it predicts improvements in OCD symptoms. The limited contribution of therapeutic alliance on positive treatment outcomes in the present study concurs with previous research that did not find associations between overall therapeutic alliance rated early in treatment by patients (15–17) or therapists (15, 18) and treatment outcome. Our findings indicated that only 2% of the improvement in OCD symptoms was accounted for by therapeutic alliance. A very large increase in strength of therapeutic alliance was needed to achieve a clinically meaningful decrease in post-treatment OCD symptom severity. The strength of the therapeutic alliance in our sample was high, making a relatively large increase in strength almost impossible to achieve, despite sufficient variability in therapeutic alliance scores. The finding of strong a therapeutic alliance is consistent with previous research (15, 16, 22), suggesting that therapeutic alliance might be necessary in the treatment of OCD, but not sufficient to bring about positive therapeutic outcomes. If a patient and therapist fail to build a strong therapeutic alliance, perhaps that risks failure of treatment. Focusing on patients with very weak therapeutic alliances (scores <2 SD from the mean), we found that the four patients with very weak therapeutic alliances from the patients perspective had a mean post-treatment OCD severity score that was 3 points above the mean of the remaining group, while the six patients with weaker therapeutic alliance from therapist's perspectives had a mean post-treatment OCD severity score that was 6 points above the mean of the remaining group. Yet, these differences in means were not statistically significant [WAI-c: *t*_(185)_ = 0.750, *p* = *0.454*, mean difference = 3.05, 95% CI (−4.97, 11.06); WAI-t: *t*_(185)_ = 1.736, *p* = *0.084*, mean difference = 5.75, 95% CI (−0.079, 12.29)], which may be due to the small sample sizes.

Compared to the present study, some previous research found larger effect sizes for the association between early overall patient-rated (12, 18, 19) or therapist-rated (17) therapeutic alliance and treatment outcome. However, Keijsers (18) only found significant effect sizes for improvement in obsessive fears, and not for improvement in compulsive behavior. There are a few differences between these previous studies and the present study that may explain the discrepancy in findings: differences in measurements to assess therapeutic alliance (the Barret Lennard Relationship Inventory [RI: (33)] by Hoogduin (17) and Keijsers (18), a relationship measure that assesses empathy, positive regard, incongruence and negative regard), in measurements to assess treatment outcome (the Maudsley Obsessional Compulsive Inventory for compulsive behavior [MOCI: (34)] and the Anxiety Discomfort Scale for obsessive fear (35) by Keijsers (18), and in populations (e.g. exclusion of patients with a major depressive disorder by Keijsers (18)) and exclusion of patients already treated with EX/RP by Simpson (12) and Maher (19)). Further, our effect sizes were smaller compared to previous studies who investigated the association between treatment outcome and therapeutic alliance assessed mid-treatment (17, 20). However, as treatment progresses, the confounding impact of prior symptom change on the alliance-outcome association increases; that is, therapeutic interventions that occur in the weeks prior to assessing therapeutic alliance may result in symptom improvement, which may increase the therapeutic alliance (36, 37). Therefore, the results of these previous studies need to be interpreted with caution.

Patient's and therapist's agreement on the strength of therapeutic alliance was low. This is consistent with previous literature, reporting weak to moderate correlations between patient and therapist ratings of therapeutic alliance (38–42). This suggests that one cannot take a patient's or therapist's perspective alone as the sole valid description of the therapeutic alliance, both in research and clinical practice. Patients and therapists may use a different frame of reference for rating the therapeutic alliance. Perhaps therapists rate the therapeutic alliance based on theoretical knowledge and relative to alliances with previous patients, whereas patients may rate the therapeutic alliance based on experiences with medical professionals, friends or family members. Therapists should be aware that their perspective on the strength of the therapeutic alliance may differ from the view of their patients.

When considering the different domains of therapeutic alliance, two domains significantly predicted OCD symptom severity after treatment: Goal and Task alliance. This finding is consistent with that of previous research that reported significant associations between Task alliance (16) or Task/Goal alliance (22) and post-treatment OCD symptom severity. These facets of alliance reflect the amount of agreement between the patient and therapist about the goals the patient will work toward in therapy and the extent to which they mutually agree on the tasks that need to be done to reach these goals. In the initial phase of OCD treatment, setting of goals, explaining the treatment rationale and formulating tasks is one of the core components, thereby possibly amplifying Goal and Task alliance. In other words, it could be argued that the specific therapy techniques in the treatment of OCD contribute to the therapeutic alliance. However, although the Goals and Task domains of therapeutic alliance showed statistically significant effects in our sample, none of the domains of therapeutic alliance showed clinically meaningful effect sizes.

In the context of a wider therapeutic alliance literature base, the effect sizes in previous research examining the impact of therapeutic alliance in CBT outcomes are quite diverse. In the treatment of anxiety disorders, two systematic reviews revealed substantial variability across studies (43, 44). Luong et al. (44) concluded that due to the inconsistencies across the studies, the impact of therapeutic alliance on CBT outcomes for anxiety disorders could not be established. In the treatment for eating disorders, a meta-analysis showed that therapeutic alliance was not related to subsequent symptom change in the treatment with CBT (45). In the treatment for depression, however, a meta-analysis showed that the therapeutic alliance was related to CBT outcomes, with an overall mean effect size of *r* = 0.26 (46). This may indicate that the therapeutic alliance has differential effects across diagnostic populations treated with CBT, consequently highlighting the importance of considering disorders separately when examining the alliance-outcome association, even within the context of CBT.

Several strengths and limitations should be taken into account when interpreting the results. Strengths include the assessment of therapeutic alliance from the therapist's perspective in addition to the patient's perspective, and the relatively large sample. A few limitations should be acknowledged as well. First, therapeutic alliance was measured at a single point in time, therefore, we were unable to examine the potential bi-directional alliance-outcome association; that is, post-treatment symptom improvement might result from the fact that positive therapeutic alliances generate symptom change, because symptom improvement strengthens therapeutic alliances, or because they mutually influence each other [e.g. (47)]. Indeed, Strauss (2018) examined alliance over time and suggested that it is more likely that therapeutic alliance is a *consequence* of OCD symptom change instead of a *predictor*. Future research should repeatedly assess therapeutic alliance since it would help to understand the direction of the relationship between potential changes in alliance and treatment outcome. Second, therapeutic alliance and OCD symptom severity were not assessed at the same time point, therefore, we were unable to take the potential confound between early symptom change and therapeutic alliance into account. Third, we included only one measure of treatment outcome, i.e. post-treatment symptom severity, while research has shown that treatment reduces OCD symptoms and improves quality of life (48, 49). Perhaps therapeutic alliance relates differently to symptom change than to quality of life. Thus, our results might not be generalizable to other measures of outcome. Fourth, we did not collect observer ratings of therapeutic alliance, therefore, we were unable to examine the predictive value of independent assessor-ratings of therapeutic alliance and treatment outcome. Although a large meta-analysis showed no differences between ratings of patients, therapists and observers and their ability to predict outcome (30), a systematic review investigating the alliance-outcome association in anxiety disorders showed that observer ratings were more related to outcome than both patient and therapist ratings (44). Currently, there are no data on the predictive value of observer-ratings of alliance on OCD treatment outcome. To develop a full picture of the impact of therapeutic alliance in the treatment of OCD, future studies could take observer-ratings of therapeutic alliance into account.

A possible concern is that we combined patients receiving different treatments. Although all treatments were cognitive behavior therapies for OCD, IBA-treatment differed from the other treatment conditions with regard to focus on specific treatment elements. However, a sensitivity analysis showed comparable effect sizes to the main analysis if patients who received IBA-treatment were not included in the analysis. Further, it could be argued that the small effect sizes in our sample were due to the fact that ~30% of our therapists sample consisted of less experienced therapist, i.e. master's students of clinical psychology. Previous research showed that therapist variability was associated with outcome, whereas patient variability was not (22, 50, 51), demonstrating the importance of therapists in building therapeutic alliances. If the extent to which therapists form alliances depends on the level of experience of therapists, this could influence the alliance-outcome association. However, a *post-hoc* analysis showed no statistical differences between experienced and trainee therapists in the strength of therapeutic alliance as rated by patients [*t*_(172)_= −0.740, *p* = 0.460]. Additionally, a sensitivity analysis did not show higher effect sizes if less experienced therapists were not included in the analysis. Due to the variability in therapist's experience in our sample, and due to the fact that our sample included a broad range of patients who were treated in different clinical settings, our results may be generalized beyond the current sample.

In summary, the present study adds to current knowledge on the predictive role of therapeutic alliance in CBT for OCD. Its results showed that the strength of therapeutic alliance contributes to OCD treatment outcome, though to a limited extent. Therapeutic alliance seems necessary in the treatment for OCD, but not sufficient to bring about therapeutic change. Therapists should deliver the core components of OCD treatment protocols, in the context of a strong therapeutic alliance, to maximize treatment outcome.

## Data Availability Statement

The original contributions presented in the study are included in the article/supplementary material, further inquiries can be directed to the corresponding author.

## Ethics Statement

The studies involving human participants were reviewed and approved by VU-University Medical Centre's Ethical Review Committee (Amsterdam, Netherlands) and METiGG (Utrecht, Netherlands). The patients/participants provided their written informed consent to participate in this study.

## Author Contributions

PO and AB designed and directed the EX/RP-RCT. HV, PO, and AB designed and directed the IBA-RCT. NW and AH performed the statistical analyses. NW took the lead in writing the manuscript. All authors contributed to conception of the present study, manuscript revision, read, and approved the submitted version.

## Funding

The IBA-RCT was partly funded by a grant from ZonMw (grant-number 100003032) and by a grant from Netherlands Foundation for Mental Health (grant-number 6351).

## Conflict of Interest

The authors declare that the research was conducted in the absence of any commercial or financial relationships that could be construed as a potential conflict of interest.

## Publisher's Note

All claims expressed in this article are solely those of the authors and do not necessarily represent those of their affiliated organizations, or those of the publisher, the editors and the reviewers. Any product that may be evaluated in this article, or claim that may be made by its manufacturer, is not guaranteed or endorsed by the publisher.
